# The submental flap: Be wary

**DOI:** 10.1002/ccr3.5260

**Published:** 2022-01-07

**Authors:** Courtney B. Shires, Merry Sebelik

**Affiliations:** ^1^ West Cancer Center Germantown Tennessee USA; ^2^ Emory University Atlanta Georgia USA

**Keywords:** head and neck reconstruction, submental flaps

## Abstract

The submental island flap (SIF) is as an alternative to free flaps in head and neck reconstruction. 10 patients underwent submental flaps. All ten patients suffered failure of SIF as the definitive reconstructive procedure. Despite comparing favorably to free tissue transfer in published reports, our SIF had high failure rate.

## Graphical abstract

Despite comparing favorably to free tissue transfer in published reports, submental island flap in this series had a high failure rate, and the need for secondary procedures negated any cost or time savings.

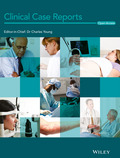

## 1 INTRODUCTION

Oral cavity cancer is the sixth most common cancer worldwide, comprising 30% of all H&N cancers.^1^ Surgery is considered the gold standard to achieve tumor control.^2^ The traditional surgery has been the use of microvascular free flaps, albeit at higher costs, and hospital length of stay than alternatives. Other head and neck resections result in tissue deficits, such as after parotidectomy. The submental island flap (SIF) has emerged as an alternative over the more costly and lengthy free flap, for oral cavity and other head and neck defects.

The first description of the SIF was by Martin and colleagues in 1993.^3^ They described it as a reliable alternative to the more traditional free flap procedure and as a means of reconstruction after oncologic procedures of the head and neck.^4^ Arising deep to the submandibular gland, the submental artery, a reliable branch of the facial artery, is the main contributor to the SIF.^4^, ^5^, ^6^ At the mylohyoid, the artery either continues deep (70%) or superficial (30%) to the anterior belly of the digastric muscle, terminating at the mandibular symphysis.^6^, ^7^ Up to 4 cutaneous perforators have been described in other SIF studies^7^; however, cadaveric studies have only been able to consistently find 1 reliable perforator to supply the skin paddle.^8^ Also, the submental vein has been found to be the primary vessel for venous drainage of the SIF.^9^ With decreased hospitalizations and shorter operating times, the SIF transformed into a plausible and effective alternative to traditional free flap reconstruction.^5^ However, even with its relatively consistent anatomy and large number of positive surgical outcomes, the SIF has had its fair share of complications.

This study seeks to analyze and explain the various post‐op complications surrounding submental island flaps. It will estimate the potential impact submental flaps may have relative to the traditional method of using free flap reconstruction, once post‐op complications are taken into consideration. It will also serve as a warning to new reconstructive surgeons who consider using a submental flap, as opposed to the more traditional free flap.

## 2 METHODS AND MATERIALS

This retrospective case series with chart review includes 10 consecutive patients that underwent SIF reconstruction following various head and neck procedures by 2 different physicians at a single care facility between November 2016 and April 2018. These surgeons were newly out of fellowship training and embarking on their first attending surgeon employment. Inclusion criteria were adults with a diagnosis of malignancy of the head and neck undergoing surgery with reconstruction using SIF that then went on to fail SIF reconstruction. Demographics and preoperative risks were collected. Data were gathered regarding the type of procedure performed. Postoperative variables and wound dehiscence were recorded.

## 3 RESULTS

Ten total patients underwent submental flaps between 2016 and 2018 (Table 1). Five were female, and 5 were male. Age of patients ranged from 33 to 85, with an average age 60.7 years. Only 2 patients were smokers. Four patients had hypertension, and one had diabetes. Six of the patients had no comorbidities. Nine of the patients had simultaneous neck dissection. None of the patients had prior chemotherapy or radiation. The defects requiring reconstruction were widely varied.

**TABLE 1:** First 10 consecutive submental flaps performed at one institution

Patient	Sex	Age	Comorbidities	Initial staging	Tobacco use	Simultaneous neck dissection	Previous radiation or chemotherapy?	Outcome	Need for second trip to OR	Defect
1	F	61	None	T3N0M0	Yes	Yes	No	Aborted due to pathologic nodes in submental area and Free Flap next day	Yes	Composite resection of right floor of mouth, right ventral tongue partial glossectomy, and right marginal mandibulectomy
2	F	33	None	T2N0M0	No	No	No	Residual postauricular defect that needed cervicofacial rotational flap reconstruction	Yes	Parotid defect
3	M	56	None	T2N0M0	Yes	Yes	No	Congested and debulked	Yes	FOM/ventral tongue
4	F	85	DM, HTN	T2N0M0	No	Yes	No	Performed a submental island flap. Later it was noted that the submental vein drained into the external jugular system	Yes	Tongue/RMT
5	F	62	HTN	T1N0M0	No	Yes	No	Aborted and did alloderm	No	FOM
6	M	59	HTN	T2N0M0	No	Yes	No	Necrotic and debulked	Yes	Partial glossectomy
7	M	67	None	T1N0M0	No	Yes	No	Congested and debulked	Yes	Buccal mucosa
8	M	53	None	T2N0M0	No	Yes	No	Aborted and did STSG	No	Ventral tongue/FOM
9	F	73	HTN	T1N0M0	No	Yes	No	Necrotic and debulked	Yes	Ventral tongue/FOM
10	M	58	None	T1N0M0	No	Yes	No	Necrotic and debulked	Yes	Buccal mucosa

All ten patients suffered failure of the SIF as the definitive reconstruction. Eight of ten patients required a second procedure in the operating room. Three of ten patients received an intraoperative change in reconstruction plan, aborting the SIF during the initial procedure. Patient 1, a 61‐year‐old woman and tobacco user, had her SIF reconstruction aborted due to pathologic nodes in the submental area which were not present on her preoperative imaging studies (PET and CT). She subsequently underwent a free flap reconstruction the following day. Patient 5, a 62‐year‐old woman with hypertension, had her SIF aborted because of poor venous flow. Instead, she had an acellular dermal matrix allograft placed. Patient 8, 53‐year‐old man with no comorbidities, had his SIF reconstruction aborted due to poor blood supply to the island graft. He then underwent a split‐thickness skin graft (STSG) the same day.

Six of the 10 patients had initial placement of the SIF and further debridement at a second OR sitting. Three of those had venous congestion, and 3 of those were due to necrosis from poor arterial supply. One patient noted survival of a portion of the SIF for a parotid defect but needed a subsequent cervicofacial rotation flap for closure of the remaining defect.

## 4 DISCUSSION

Although the submental flap is relatively thin, easy‐to‐harvest, and typically well‐vascularized, it does have complications.^10^ Our single‐institution series varied from the literature with 100% failure rate. Our two reconstructive surgeons each completed a 1‐year fellowship in head and neck cancer reconstruction. They each had performed over 100 free flaps in their fellowship training, but had both performed less than 5 submental flaps in their training.

Chow et al. reported partial loss of 2 out of 10 flaps in their 2007 study, while Merten et al. reported loss of 1 flap in 11 nonirradiated patients in their 2002 study.^11^, ^12^ In a series of SIF performed in 2018 by Faisal et al., 2 complete and 3 partial flap losses were recorded.^10^ The authors mentioned that they avoided the SIF if the neck had been previously irradiated, with Taghinia et al. reporting that preoperative radiotherapy was the most consistent finding in those who suffered flap loss.^13^

All 10 of our patients had disease‐free necks preoperatively, with stage N0 based on preoperative CT and/or PET. Nine of our 10 patients required simultaneous neck dissection. When a neck dissection is needed during a procedure where SIF is planned, the reconstructive surgeon should have a careful discussion with the resecting head and neck surgeon so that the facial artery or vein is not ligated during the neck dissection. In the circumstance that the vein or artery is injured, using that side of the neck for the SIF is not recommended, and the submental flap should be based on the contralateral side.

Three of the patients were noted to have venous congestion, requiring second trip to the OR. The submental vein has been found to be the primary venous drainage of the flap, but in one of our cases, the submental vein was noted to drain into the external jugular system during the bring‐back procedure. The external jugular system had been ligated during the initial procedure. Perhaps this could have been avoided with an earlier identification of the anatomy. A different mode of reconstruction could have been undertaken during the initial procedure.

Three of the patients were noted to have necrotic SIF from lack of blood supply. Studies have shown only one reliable perforator of the SIF, which is much smaller than the perforators of the work‐horse anterolateral thigh free flaps and radial forearm free flaps. The size difference for vessel handling can be a potential technical challenge.

Our poor SIF results were independent of the defect site. We used SIF for soft tissue defects resulting from composite resection of mandible/tongue/floor of mouth; as well as defects of oral tongue; retromolar trigone; buccal mucosa; floor of mouth; and parotid. Sittitrai and colleagues concluded that the SIF is reliable, is suitable for oral tongue reconstruction, and had a lower complication incidence when compared to the radial forearm free flap.^14^ While there is an abundance of support for free flaps and the success is >95% in the literature, there are also as much positive data on the SIF.

Our reconstructive surgeons had a greater than 90% survival rate when performing free flaps and a 0% success rate when using SIF. Thus, technical and training factors were examined. In typical head and neck surgical oncology fellowships, free flaps are a far more common form of reconstruction than SIF. Regenbogen and several others have acknowledged that commonly recommended interventions, like restricting high‐complexity operations to experienced surgeons and additional trainings for inexperienced surgeons would lead to an improvement in outcomes.^15^ In our hands, free tissue transfers have superior outcomes compared with pedicled flaps due to our experience and knowledge of free flaps and our deficiency of these with SIF.

Studies have shown less cost with pedicled flaps than free flaps. However, 70% of our SIF patients required second trips to the operating room during their initial stay, compared with 5% of our free flap patients. In our hands, patients who underwent SIF did not experience decreased cost, length of stay, and operative time compared with free flaps, as reported in other series.^14^, ^16^, ^17^, ^18^

As Zhou and colleagues reiterate, in regard to intraoperative factors, surgical technique is regarded as the most important component of free flap success.^19^, ^20^ In their very own study, Zhou had two surgeons perform the microvascular anastomoses in the free flap reconstructions for his study, with each having been in practice 5 years or more.^21^, ^22^ Such experiential and technical rigor has not been analyzed in SIF outcome literature, perhaps because SIF is may be viewed as a simpler procedure.

Three of our ten patients received an intraoperative change in reconstruction plan, abandoning the SIF during the initial procedure. One patient was not reconstructed that day and underwent a free flap reconstruction the following day. A second patient had an acellular dermal matrix allograft placed the same day. A third patient underwent a split‐thickness skin graft (STSG) the same day. In retrospect, given our team's great success with free flap reconstruction and our dismal success with SIF, we should be prepared for free flap reconstruction in any patient planned for SIF on the same day as their resection. This can likely avoid return to the operating room for a second procedure.

## 5 CONCLUSION

Despite their promising potential and numerous cases with positive outcome results, submental flaps are still open to failure. In our series, surgeon experience may have been more influential in outcomes than patient factors such as age, type of defect, history of radiation, tobacco use, or other comorbidities. In our institution, a free flap is preferable and has lower risk of complications compared with a pedicle flap, and the pedicled flap is the backup secondary option when a free flap is lost.

## CONFLICT OF INTEREST

The authors report no relevant financial disclosures related to this current work.

## AUTHOR CONTRIBUTIONS

Each author contributed to development of the project, data collection, writing of the manuscript, editing, and submitting the manuscript.

## ETHICAL APPROVAL

All issues related to ethics were taken into consideration throughout the study design and proposal and implemented during the research study itself. Informed consent was obtained, beneficence was made a top priority, and respect for confidentiality and privacy were upheld during the study and its various analysis and information assertion components. The patients agreed to allow the authors to publish the case and included images.

## CONSENT

Published with written consent of the patients.

## Data Availability

Data are available upon request.

## References

[1] Vigneswaran N , Williams MD . Epidemiologic trends in head and neck cancer and aids in diagnosis. Oral Maxillofac Surg Clin North Am. 2014;26(2):123‐141. 24794262 10.1016/j.coms.2014.01.001 PMC4040236

[2] Almadori G , Rigante M , Bussu F , et al. Impact of microvascular free flap reconstruction in oral cavity cancer: our experience in 130 cases. Acta Otorhinolaryngol Ital. 2015;35(6):386‐393. 26900243 10.14639/0392-100X-919 PMC4755058

[3] Barton BM , Riley CA , Pou JD , Hasney CP , Moore BA . The submental island flap is a viable reconstructive option for a variety of head and neck ablative defects. Ochsner J. 2018;18(1):53‐58. 29559870 PMC5855423

[4] Martin D , Pascal JF , Baudet J , et al. The submental island flap: a new donor site. Anatomy and clinical applications as a free or pedicled flap. Plast Reconstr Surg. 1993;92 5:867‐873. 8415968

[5] Paydarfar JA , Patel UA . Submental island pedicled flap vs radial forearm free flap for oral reconstruction. Arch Otolaryngol Head Neck Surg. 2011;137(1):82‐87. 21242553 10.1001/archoto.2010.204

[6] Vural E , Suen JY . The submental island flap in head and neck reconstruction. Head Neck. 2000;22:572‐578. 10941158 10.1002/1097-0347(200009)22:6<572::aid-hed5>3.0.co;2-k

[7] Sterne GD , Januszkiewicz JS , Hall PN , Bardsley AF . The submental island flap. Br J Plast Surg. 1996;49(2):85‐89. 8733345 10.1016/s0007-1226(96)90078-8

[8] Abouchadi A , Capon‐Degardin N , Patenôtre P , Martinot‐Duquennoy V , Pellerin P . The submental flap in facial reconstruction: advantages and limitations. J Oral Maxillofac Surg. 2007;65(5):863‐869. 17448834 10.1016/j.joms.2006.05.063

[9] Ferrari S , Copelli C , Bianchi B , et al. The submental island flap: pedicle elongation and indications in head and neck reconstruction. J Craniomaxillofac Surg. 2014;42:1005‐1009. 24642090 10.1016/j.jcms.2014.01.025

[10] Faisal M , Adeel M , Riaz S , et al. The submental island flap in head and neck cancer. Ann Maxillofac Surg. 2018;8(2):287‐291. 30693247 10.4103/ams.ams_225_18 PMC6327798

[11] Chow TL , Chan TT , Chow TK , Fung SC , Lam SH . Reconstruction with submental flap for aggressive orofacial cancer. Plast Reconstr Surg. 2007;120:431‐436. 17632345 10.1097/01.prs.0000267343.10982.dc

[12] Merten SL , Jiang RP , Caminer D . The submental artery Island flap for head and neck reconstruction. ANZ J Surg. 2002;72:121‐124. 12074063 10.1046/j.1445-2197.2002.02318.x

[13] Taghinia AH , Movassaghi K , Wang AX , Pribaz JJ . Reconstruction of the upper aerodigestive tract with the submental artery flap. Plast Reconstr Surg. 2009;123:562‐570. 19182614 10.1097/PRS.0b013e3181977fe4

[14] Sittitrai P , Reunmakkaew D , Srivanitchapoom C . Submental island flap versus radial forearm free flap for oral tongue reconstruction: a comparison of complications and functional outcomes. J Laryngol Otol. 2019;133(5):413‐418. 31006409 10.1017/S0022215119000744

[15] Regenbogen SE , Greenberg CC , Studdert DM , et al. Patterns of technical error among surgical malpractice claims. Ann Surg. 2007;246(5):705‐711. 17968158 10.1097/SLA.0b013e31815865f8

[16] Jørgensen MG , Tabatabaeifar S , Toyserkani NM , et al. Submental island flap versus free flap reconstruction for complex head and neck defects. Otolaryngol Head Neck Surg. 2019;161(6):946‐953. 31500500 10.1177/0194599819875416

[17] Pradhan P , Samal S , Samal DK , et al. Submental island flap reconstruction for carcinoma of the oral cavity: experience in 30 cases. World J Otorhinolaryngol Head Neck Surg. 2019;5(2):65‐70. 31334483 10.1016/j.wjorl.2018.03.007 PMC6617528

[18] Forner D , Phillips T , Rigby M , et al. Submental island flap reconstruction reduces cost in oral cancer reconstruction compared to radial forearm free flap reconstruction: a case series and cost analysis. J Otolaryngol Head Neck Surg. 2016;45:11. 26846792 10.1186/s40463-016-0124-8 PMC4743171

[19] Valentini V , Cassoni A , Marianetti TM , et al. Diabetes as main risk factor in head and neck reconstructive surgery with free flaps. J Craniofac Surg. 2008;19(4):1080‐1084. 18650736 10.1097/SCS.0b013e3181763531

[20] Spiegel JH , Polat JK . Microvascular flap reconstruction by otolaryngologists: prevalence, postoperative care, and monitoring techniques. Laryngoscope. 2007;117:485‐490. 17334309 10.1097/MLG.0b013e31802d6e66

[21] Zhou W , Zhang WB , Yu Y , et al. Risk factors for free flap failure: a retrospective analysis of 881 free flaps for head and neck defect reconstruction. Int J Oral Maxillofac Surg. 2017;46:941‐945. 28416356 10.1016/j.ijom.2017.03.023

[22] Sanati‐Mehrizy P , Massenburg BB , Rozehnal JM , et al. Risk factors leading to free flap failure: analysis from the national surgical quality improvement program database. J Craniofac Surg. 2016;27(8):1956‐1964. 28005734 10.1097/SCS.0000000000003026

